# Association of *KCNJ6* rs2070995 and methadone response for pain management in advanced cancer at end-of-life

**DOI:** 10.1038/s41598-022-21180-w

**Published:** 2022-10-19

**Authors:** Deniz Ozberk, Alison Haywood, Heidi G. Sutherland, Chieh Yu, Cassie L. Albury, Mathew Zunk, Rani George, Phillip Good, Lyn R. Griffiths, Janet Hardy, Larisa M. Haupt

**Affiliations:** 1 School of Pharmacy and Medical Sciences, Griffith University, Gold Coast, Australia; 2 Mater Research Institute, The University of Queensland, Brisbane, Australia; 3 Stem Cell and Neurogenesis Group, Genomics Research Centre, Centre for Genomics and Personalised Health, Queensland University of Technology, 60 Musk Ave, Kelvin Grove, Brisbane, QLD 4059 Australia; 4 Department of Cell and Tissue Biology, University of California, San Francisco, USA; 5 Cancer Trials Unit, Division of Cancer Services, Metro South Health, Brisbane, Australia; 6 Department of Palliative Care, St Vincent’s Private Hospital, Brisbane, Australia; 7 Department of Palliative and Supportive Care, Mater Health, Brisbane, Australia

**Keywords:** Cancer, Genetics, Oncology

## Abstract

Opioids are the therapeutic agents of choice to manage moderate to severe pain in patients with advanced cancer, however the unpredictable inter-individual response to opioid therapy remains a challenge for clinicians. While studies are few, the *KCNJ6* gene is a promising target for investigating genetic factors that contribute to pain and analgesia response. This is the first association study on polymorphisms in *KCNJ6* and response to methadone for pain management in advanced cancer. Fifty-four adult patients with advanced cancer were recruited across two study sites in a prospective, open label, dose individualisation study. Significant associations have been previously shown for rs2070995 and opioid response in opioid substitution therapy for heroin addiction and studies in chronic pain, with mixed results seen in postoperative pain. In this study, no associations were shown for rs2070995 and methadone dose or pain score, consistent with other studies conducted in patients receiving opioids for pain in advanced cancer. There are many challenges in conducting studies in advanced cancer with significant attrition and small sample sizes, however it is hoped that the results of our study will contribute to the evidence base and allow for continued development of gene-drug dosing guidelines for clinicians.

## Introduction

Cancers are among the leading causes of morbidity and mortality worldwide, and pain is the most debilitating symptom associated with cancer that can significantly impact quality of life^1^. Opioids are the therapeutic agents of choice to manage moderate to severe pain in patients with cancer in end-of-life^2^, however the unpredictable inter-individual response to opioid therapy remains a challenge for clinicians^1^. Pharmacogenetics has been shown to be a promising approach to tailor treatment to an individual’s genetic profile as cancer patients with a more favourable genetic background have been shown to respond better to opioids with a lower dose and fewer side effects^3,4^.

The *KCNJ6* gene has been shown to be a promising target for investigating the genetic factors that contribute to pain and analgesia response^5^. *KCNJ6* encodes for potassium inwardly rectifying channels, subfamily J, member 6 (GIRK2, Kir3.2). G-protein coupled inwardly rectifying potassium (GIRK) channels are activated by heterotrimeric G_i/o_ proteins after stimulation of opioid receptors by endogenous or exogenous opioids. This causes an efflux of potassium ions which hyperpolarize the membrane potential and dampen neuronal excitability thus limiting nociceptive transmission^6^. Due to the crucial function of GIRK channels in the therapeutic effect of opioids, it has been suggested that future analgesic agents may be developed to directly target GIRK channels^7–9^.

Genetic variation in the *KCNJ6* gene has been shown to influence opioid response^5^, where the G allele of the A1032G (rs2070995) polymorphism was associated with increased opioid requirements in postoperative pain^10^ and chronic pain^11^, however not all studies have shown this association^12,13^. In opioid substitution therapy for former heroin addicts, increased methadone dose requirements and reduced withdrawal effects were associated with homozygous carriers of the A allele^11^.

Methadone is usually prescribed by palliative care specialists as a second choice opioid, for switching from another opioid to improve analgesia and/or reduce adverse effects, and in difficult pain control scenarios including neuropathic pain syndromes and hyperalgesic states^14^. Dosing is challenging, with vigilant dose initiation, adjustment and monitoring required^15^. Methadone is a long acting synthetic opioid and has antagonist activity at the N-methyl-D-aspartate (NMDA) receptor in addition to its activity at the μ opioid receptor^16^. Despite the many advantages of utilising methadone (low cost, rapid onset of effect, high oral bioavailability and lack of active metabolites), with some suggesting that it may even be effective as a first-line opioid in the management of cancer pain, its prescription is restricted to specialists due to its complex pharmacokinetics and variability in dose requirements and side effects experienced between patients^16^.

The aim of this study was to determine any association of *KCNJ6* rs2070995 with opioid requirements and therefore contribution to inter-individual variability in response to methadone for pain management in patients with advanced cancer.

## Materials and methods

### Study participants and procedures

Adult patients with advanced cancer who were treated at the oncology and palliative care services of the Mater Adults Hospital (MAH) and St Vincent’s Private Hospital (SVPH) in Brisbane between 2013 and 2016 were eligible for inclusion in an open-label dose individualisation study on the use of methadone in pain management. Patients who were age ≥ 18 years, able to read and understand the patient information sheet, able to provide written consent, and willing to provide blood and saliva samples were enrolled in the study. Exclusion criteria included those patients with oral mucositis, infection, or xerostomia. A sample size of 50 participants, providing two to four samples, was determined to be the minimum number necessary to generate satisfactory estimates of the structural parameters (clearance and volume of distribution) and the variance parameters (interindividual and inter-occasion variability) for non-linear mixed effect modelling (population pharmacokinetic modelling) for the dose individualisation study. Patient characteristics and clinical data including type of cancer, liver and renal function, and methadone dose were recorded. Pain intensity was assessed using the Brief Pain Inventory^17^, where patients were required to rate their pain from 0 to 10, with a score of 0 representing “no pain” and 10 representing “pain as bad as you can imagine”. Pain scores were recorded each time blood and saliva were collected, and at a time convenient to the participant. Methadone was administered via the oral route twice daily with dosing titrated according to patient need by the palliative care specialist. The study was granted ethics approval by MAH (#HREC/13/MHS/103) and SVPH (#HREC/13/15) Human Research Ethics Committees.

### Genotyping

Genomic DNA (gDNA) was extracted from whole blood collected into EDTA tubes using an in-house salting-out method^18^ at the Genomics Research Centre, Queensland University of Technology, Brisbane. A NanoDrop™ ND-1000 spectrophotometer (ThermoFischer Scientific Inc., Waltham, MA, USA) was used to measure DNA concentration and purity before dilution to 15–20 ng/μL and storing as stock gDNA at 4 °C. Genotyping of *KCNJ6* (rs2070995, 1032A > G) was conducted via pyrosequencing with primers designed using Pyromark Assay Design software (QIAGEN): 5′TTGACAATGGACCCCAACA, 5′TGGTTATGGCTACCGGGTCA (biotinylated) and sequencing primer 5′TTAAGAGAAGAATAATTCCC. Pyrosequencing was performed on a QSeq platform (BioMolecular Systems) using Pyromark Gold Q24 reagents (QIAGEN). Sequencing traces were analysed with QSeq software, version 2.1.3 (BioMolecular Systems). All genotyping was conducted by investigators blinded to sample identity.

### Statistical analysis

Clinical data are described as mean ± standard deviation (SD) or medians and interquartile ranges, as appropriate for continuous measures. Nominal variables are described as frequencies and percentages. Regression analysis was used to examine whether the outcomes of methadone dose or pain score were dependent on any patient characteristics not related to *KCNJ6* rs2070995 genotype, including gender, age, body mass index (BMI), liver and kidney function. Deviation of Hardy–Weinberg equilibrium was determined by comparing the observed genotype frequencies with the expected values using the chi-square (χ^2^) test. The Kruskal–Wallis H test was used to determine whether genotypes were associated with methadone dose or pain score. Methadone dose and pain scores were averaged across all samples for participants providing multiple samples. χ^2^ analysis was used to determine significant associations for high pain score (> 3/10) and high methadone dose (> 10 mg/day), when outcomes were categorised. The adequacy of each statistical test was assessed by examining residuals for heterogeneity and normality. Significance was considered if *p* < 0.05. The observed minor allele frequency (MAF) was compared to the MAF for relevant populations reported for ALFA and 1000Genomes in dbSNP (National Center for Biotechnology Information)^19^. Data was analysed using IBM SPSS Statistics for Windows, version 26.0 (Armonk, NY: IBM Corp).

### Ethics approval and consent to participate

This study was performed in line with the principles of the Declaration of Helsinki. Approval was granted by the Human Research Ethics Committees at Mater Health Services (# HREC/13/MHS/103), St Vincent’s Health and Aged Care (#HREC/13/15) and Griffith University (#PHM/17/13/HREC). Informed consent was obtained from all individual participants included in the study.

## Results

Of the 54 adult patients with advanced cancer recruited, complete genotyping data and pain scores were available for 46 participants. Methadone was administered orally, and the prescribed dose ranged from 2.5–50 mg twice daily. Patient characteristics including age, gender, BMI and cancer type are shown in Table 1. The median (IQR) methadone daily dose and patient reported pain score (on a scale of 0–10) for our population was 11.3 ± 13.9 mg and 3.9 ± 3.2, respectively. No patient characteristics were found to significantly determine the outcomes of methadone dose or pain score, including age, gender, height, weight, BMI, liver and renal function (Supplementary Table 1).

**Table 1:** Patient characteristics.

Patient characteristic (n = 46)	Value	
Age (years)	Mean ± SD (range)	60.6 ± 13.7 (32–83)
Weight (kg)		68.4 ± 16.9 (36–118)
BMI (kg/m^2^)		24.9 ± 5.1 (16–38.5)
Gender* n* (%)	Female	27 (58.7)
	Male	19 (41.3)
Cancer type *n* (%)	Breast	8 (17.4)
	Colorectal	8 (17.4)
	Cervical	6 (13.0)
	Lung	5 (10.9)
	Mesothelioma	4 (8.7)
	Endometrial	3 (6.5)
	Pancreatic	3 (6.5)
	Prostate	2 (4.3)
	Multiple myeloma	2 (4.3)
	Gallbladder	1 (2.2)
	Bladder	1 (2.2)
	Liver	1 (2.2)
	Periauricular squamous cell carcinoma	1 (2.2)
	Skin cancer	1 (2.2)

Analysis for our study population identified GG (n = 26) and GA (n = 20) genotypes with no patients identified to be carrying the homozygous AA genotype. The genotype distribution was in agreement with Hardy–Weinberg equilibrium (*p* > 0.05). The MAF for this marker in European populations is reported to be 0.208 (ALFA) and 0.202 (1000Genomes)^19^, and is comparable to the observed MAF for our population (0.217). Ethnic variance is seen in rs2070995, with a study conducted in Japan observing a MAF of 0.344^10^, which is comparable to the reported MAF for East Asian populations of 0.396 and 0.361 for the ALFA and 1000Genomes databases, respectively^19^. African populations show the lowest MAF with 0.0535 and 0.0068 reported for the ALFA and 1000Genomes databases, respectively^19^.

The genotype frequencies and methadone daily dose and patient reported pain score are shown in Table 2. No significant associations were shown for methadone dose or pain score between genotypes when treated as continuous variables (*p* > 0.05). No significant association was shown between genotypes for low (≤ 3/10) or high (> 3/10) pain scores, or low (≤ 10 mg/day) or high (> 10 mg/day) methadone dose (*p* > 0.05).

**Table 2:** Methadone dose and pain score for each genotype.

Phenotype outcome measure	Genotype	Value	Statistic	*p* value
Methadone dose (mg/day) *Median* ± *IQR* (*min, max*)	GG	10.0 ± 10.0 (4.0, 58.3)	*H* = 0.002	0.965^a^
	GA	13.8 ± 19.5 (3.8, 93.3)		
Low dose (≤ 10 mg/day) *n* (%)	GG	14 (60.9)	*χ*^2^ = 0.354	0.552^b^
	GA	9 (39.1)		
High dose (> 10 mg/day) *n* (%)	GG	12 (52.2)		
	GA	11 (47.8)		
Pain score (0–10) *Median* ± *IQR* (*min, max*)	GG	3.8 ± 3.3 (0.7, 7.1)	*H* = 0.0005	0.982^a^
	GA	3.9 ± 2.8 (0, 8.0)		
Low pain score (≤ 3/10) *n* (%)	GG	12 (60.0)	*χ*^2^ = 0.174	0.676^b^
	GA	8 (40.0)		
High pain score (> 3/10) *n* (%)	GG	14 (53.8)		
	GA	12 (46.2)		

^a^*p* > 0.05 as determined by Kruskal–Wallis H test or ^b^Chi-squared test for association of methadone dose or pain score with genotype GG (n = 26) compared to GA (n = 20).

The results of our literature review on the association of *KCNJ6* rs2070995 and response to opioids for pain conditions, including chronic pain, post-operative pain and cancer pain, and response to methadone in opioid substitution therapy is summarised in Table 3. Our findings are consistent with other studies in advanced cancer, but in contrast to those studies on opioid response in opioid substitution therapy and chronic pain, with mixed results seen in postoperative pain, and will be discussed further below.

**Table 3:** Characteristics of studies on the association of *KCNJ6* rs2070995 and response to opioids.

Study reference	Therapeutic area	Study site, design and included participants	Intervention	Case numbers and MAF*	Study findings
Matic et al.^12^	Advanced cancer	238 advanced cancer patients referred to a pain consultation service due to inadequate analgesia, Netherlands	Opioids (fentanyl 73.3%, oxycodone 43%, hydromorphone 11%, morphine 5%, buprenorphine 5%) with 9% requiring ketamine as an adjuvant analgesic	GG (n = 10) GA (n = 81) AA (n = 147)	No association was found between genotypes and morphine equivalent dose (MED) or relative change in MED from baseline. No association was found for use of ketamine as an adjuvant analgesic
Oosten et al.^13^	Advanced cancer	335 moderate-to-severe cancer-related pain, Netherlands	Opioids (oxycodone, morphine, fentanyl, hydromorphone)	GG (n = 215) GA + AA (n = 120)	No association between genotypes and opioid failure, defined as rotation to another opioid or treatment with intrathecal opioids due to insufficient pain control and/or side effects, or the use of palliative sedation because of refractory symptoms associated with opioid treatment in the dying phase
Lotsch et al.^11^	Chronic pain	352 chronic pain patients treated for pain of various reasons in tertiary outpatient care, Germany	Opioids (morphine, fentanyl, buprenorphine, oxycodone, tilidine, tramadol, hydromorphone, dihydrocodeine, levomethadone, piritramide)	AA (n = 17) MAF = 0.2	AA genotype associated with a significantly higher oral MED than combined AG and GG genotypes, with no significant difference in AA genotype distribution for pain diagnoses or opioid used, and no significant difference in pain score
Margarit et al.^20^	Chronic pain	222 patients with chronic lower back pain referred for opioid prescription, Spain	Opioids (fentanyl, tramadol, oxycodone, morphine, tapentadol, buprenorphine)	AA (n = 63) AG (n = 33) GG (= 5) MAF = 0.21	Carriers of the A allele (AA and AG) were associated with a significantly higher pain intensity at the final visit and at the follow up visit (2–4 years later) than the GG genotype
Bruehl et al.^5^	Post-operative pain	311 white patients receiving opioids after total knee arthroplasty (TKA), United States	Opioids (96.4% of orders were for oral immediate release oxycodone)	MAF = 0.229	No association between genotypes and total number of oral opioid analgesic medication orders for patients undergoing TKA. It was not possible in the study to examine the number of individual analgesic medication doses actually administered or directly assess their efficacy
Nishizawa et al.^10^	Post-operative pain	129 undergoing major open abdominal surgery (mostly gastrectomy for gastric cancer and colectomy for colorectal cancer), Japan	Continuous epidural fentanyl or morphine diluted with bupivacaine. Opioids (morphine, buprenorphine, pentazocine, pethidine) and/or NSAIDS used for rescue analgesia	AA (n = 11) AG (n = 62) GG (n = 56) MAF = 0.344	AA genotype required rescue pain medication more frequently than AG and GG genotypes, with no associations for postsurgical pain ratings observed. A trending increase in oral MED was seen for AA genotypes, which was significant for female patients
Lotsch et al.^11^	Opioid substitution therapy	85 patients on methadone substitution therapy for heroin addiction, Germany	Methadone	AA (n = 4) AG (n = 12) GG (n = 69) MAF = 0.22	AA genotype had significantly higher average and maximum daily methadone doses during the first year of substitution therapy than combined AG and GG genotypes, and AA carriers lacked opioid withdrawal symptoms

*Data for MAF or genotype distribution as reported in the study.

## Discussion

Polymorphisms in *KCNJ6* have not been widely investigated^5^. This is the first association study on opioid response in patients with advanced cancer, where methadone has been used as the therapeutic intervention. Only one study has previously investigated association between methadone dose requirements and polymorphisms in *KCNJ6* (rs2070995), reporting a significant association for 85 patients on opioid substitution therapy for heroin addiction^11^.

Two studies have been conducted in patients with advanced cancer. Both studies involved participants in European populations, and consistent with our findings, no association was found for rs2070995 and opioid response^12,13^. Matic et al*.*^12^ found no association between genotypes and morphine equivalent dose (MED) or relative change in MED from baseline. Additionally, no association was found for the use of ketamine as an adjuvant analgesic. Oosten et al*.*^13^ found no association between genotypes and opioid failure—defined as rotation to another opioid or treatment with intrathecal opioids—due to insufficient pain control and/or side effects, or the use of palliative sedation because of refractory symptoms associated with opioid treatment in the dying phase.

In contrast, studies in chronic pain, Lotsch et al*.*^11^ reported the AA genotype to be associated with a significantly higher opioid requirement than combined AG and GG genotypes, and Margarit et al*.*^20^ reported that carriers of the A allele (AA and AG) were associated with a significantly higher pain intensity score than those carrying the GG genotype. Similarly, in post-operative pain, Nishizawa et al*.*^10^ reported the AA genotype to require rescue pain medication more frequently than AG and GG genotypes, with no association identified for postsurgical pain ratings. Bruehl et al*.*^5^ reported no association between genotype and total number of oral opioid analgesic medication orders for patients undergoing total knee arthroplasty. In a study conducted in patients receiving methadone maintenance therapy for heroin addiction, it was shown that homozygous carriers of the A allele required more methadone yet had fewer withdrawal symptoms than the heterozygous AG and GG genotypes^11^.

Several studies have also been conducted for different polymorphisms in *KCNJ6*. Nishizawa et al*.*^21^ investigated 27 SNPs and reported that rs2835859 may serve as a marker that predicts sensitivity to analgesia and pain. Carriers of the C allele required less postoperative fentanyl after cosmetic orthognathic surgery in a study on healthy participants (n = 355), and this finding was substantiated in a further study by the same authors of 500 healthy participants, where C allele carriers were found to have less pain perception than non-carriers, for cold pressor and mechanically-induced pain tests^21^.

Elens et al*.*^22^ investigated the association of rs6517442 and opioid requirements (morphine or remifentanil) in 34 preterm infants requiring endotracheal intubation, reporting that those with the AA genotype needed more time to reach a pain-free state after intubation than infants with the AG or GG genotypes. This finding was consistent with Margarit et al*.*^20^ and Nishizawa et al*.* 10], who also investigated rs6517442 and reported similar associations for carriers of the A allele and pain intensity^20^, or requirement for rescue analgesia^10^. The study by Matic et al*.*^12^, however, reported no associations for rs6517442. A candidate gene replication study in paediatric postoperative pain including children of African American (n = 241) and European Caucasian (n = 277) ancestry, also showed association for rs6517442, in addition to polymorphisms in rs928723, rs2211843, rs2835925, rs2835930 in the same direction for various pain phenotypes across both ethnicities in postoperative pain managed with morphine^23^.

Caution is advised when interpreting findings of our study and those reviewed for clinical application, especially when considering the heterogeneity of phenotype outcome measures across studies, which ranged from opioid dose, pain relief, the need for opioid rotation, pain intensity, number of analgesic medication orders, and the requirement for rescue analgesia. A wide variety of opioids were also used across studies as the treatment intervention, in some cases in addition to other analgesics including nonsteroidal anti-inflammatory drugs and anticonvulsants (gabapentin, pregabalin). Although we collected data over an extended period across two study sites, our sample was small and did not include any participants with the homozygous AA genotype. Further studies are needed with larger sample sizes and consistent phenotype outcome measures to provide convincing evidence that polymorphisms in *KCNJ6* contribute to inter-patient variability in opioid response in palliative care. Ethnic variance in allele frequencies in polymorphisms in *KCNJ6* may also account for the mixed results in association studies, with significant differences seen in the MAF for African, Asian and European populations^19^, highlighting the importance of taking ancestry into account when considering individual dosing considerations in the clinical setting.

The rapid growth of evidence-based gene-drug dosing guidelines and prescribing recommendations that are freely accessible online for clinicians is a promising new area for pharmacogenomics and personalised care. The Clinical Pharmacogenetic Implementation Consortium (CPIC) is an international consortium that systematically grades evidence updated in ClinGen and PharmGKB and provides genotype-based drug dosing guidelines^24^. This repository is continually updated as new studies become available making it an invaluable tool for clinicians to support future therapeutic decisions, while also expediting the translation of research findings to the clinic. In future, as more research is published, initial dosing considerations will be able to account for any significant genotype associations and response to opioids, thereby improving pain management and quality of life for patients^4^.

## Conclusion

Consistent with two other studies on opioid response in advanced cancer, our study showed no significant association for the polymorphism in *KCNJ6* rs2070995 and response to methadone for pain management. Associations have been shown for opioid response in chronic pain and opioid substitution therapy, with mixed results seen for post-operative pain, however studies are few. Further research is required before convincing evidence can show that polymorphisms in *KCNJ6* contribute to inter-patient variability in opioid response in palliative care. As the technology in pharmacogenomic testing becomes more accessible and economical, the ability for pain management therapy to be guided by precision genomic information provides a promising area for improving quality of life in palliative care.

## Supplementary Information

Supplementary Information.

## Data Availability

All data generated or analysed during this study are included in this published article (and its supplementary information files).
